# A new species of *Cintractiella* (Ustilaginales) from the volcanic island of Kosrae, Caroline Islands, Micronesia

**DOI:** 10.3897/mycokeys.42.27231

**Published:** 2018-11-06

**Authors:** M. Catherine Aime, Teeratas Kijpornyongpan, Mehrdad Abbasi, Kenneth R. Wood, Tim Flynn

**Affiliations:** 1 Purdue University, Department of Botany and Plant Pathology, West Lafayette, Indiana, USA Purdue University West Lafayette United States of America; 2 National Tropical Botanical Garden, 3530 Papalina Road, Kalaheo, HI 96741, USA Department of Botany, Iranian Research institute of Plant Protection Tehran Iran; 3 Department of Botany, Iranian Research institute of Plant Protection, Agricultural Research, Education and Extension Organization (AREEO), Tehran, Iran National Tropical Botanical Garden Kalaheo United States of America

**Keywords:** Biodiversity, phytopathogens, sedges, South Pacific, Ustilaginomycotina, 1 new taxon

## Abstract

*Cintractiella* is an unusual genus of smut fungi containing two described species that produce sori as adventitious gall-like spikelets on members of tribe *Hypolytreae* (subfam. Mapanioideae, Cyperaceae). In September 200, during a botanical expedition on the volcanic island of Kosrae located in the eastern Caroline Islands and within the Federated States of Micronesia, a specimen of *Mapaniapacifica* was collected displaying *Cintractiella*-like sori in adventitious spikelets on the host leaves. Sori were hypophyllous, occurring in groups of spikelets composed of olivaceous-brown scale-like leaves, 1–1.5 mm wide and up to 6 mm long. Microscopic comparison with the protologue and drawings of the type material of *C.lamii* show several differences in teliospore and sori characters between it and the newly collected material on *Mapania*. To our knowledge, this represents only the second known collection of any member of *Cintractiella* on vegetative organs of *Hypolytreae* and a third species for this genus and the only known smut species infecting *Mapania*, herein described as *Cintractiellakosraensis***sp. nov.**

## Introduction

There are strong correlations between the classification of smut fungi and the systematics of their host plants. For example, species of the smut genera *Anthracoidea*, *Aurantiosporium*, *Cintractia*, *Dermatosorus*, *Farysia*, *Kuntzeomyces*, *Leucocintractia*, *Moreaua*, *Orphanomyces*, *Schizonella*, *Testicularia*, *Trichocintractia* and *Ustanciosporium* exclusively infect members of Cyperaceae (Piepenbring 2001).

*Cintractiella* Boedijn, with only two known species, is an example of a smut genus that appears to be restricted to Cyperaceae, in this case wholly within the tribe *Hypolytreae*. *Cintractiellalamii* Boedijn, the type species of the genus, is only known from the locus classicus from Indonesia. The species produces sori in adventitious spikelets on leaves of a *Hypolytrum* sp. (Cyperaceae, subfam. Mapanioideae, tribe Hypolytreae). The type specimen was collected in Indonesia in 1920 and preserved in alcohol at Herb. Bogoriense (BO). Boedijn (1937) investigated the material thoroughly and described it as a new smut fungus in a new genus. Since that time, the fungus has not been recollected. Unfortunately, neither type material nor other collections of this species are available for study. The type specimen in Bogor was lost; only the empty glass vessel and label is present (Piepenbring 2001, Vánky 2003). Thus, our knowledge of this species is based on the original publication for *C.lamii* (Boedijn 1937, for a reproduction see Vánky 2013). The second species, *C.diplasiae* (Henn.) M. Piepenbr., was originally described as *Ustilagodiplasiae* Henn., on *Diplasiakarataefolia* L.C.Rich. (Hypolytreae). The type specimen was collected from Brazil and the species is also known from Venezuela on the same host species (Vánky 2003). In addition to differences in host plant and distribution, *C.diplasiae* differs from *C.lamii* in producing sori in the host inflorescences, rather than on the leaves.

In September 2009, an unusual smut fungus producing spikelets on the leaves of *Mapaniapacifica* (Hosok.) T.Koyama (*Hypolytreae*) was discovered on the island of Kosrae within the Federated States of Micronesia, herein described as a third species of *Cintractiella, C.kosraensis* sp. nov. To our knowledge, *C.kosraensis* is the only smut species known to infect a species of *Mapania.*

## Methods

**Figure 1.**
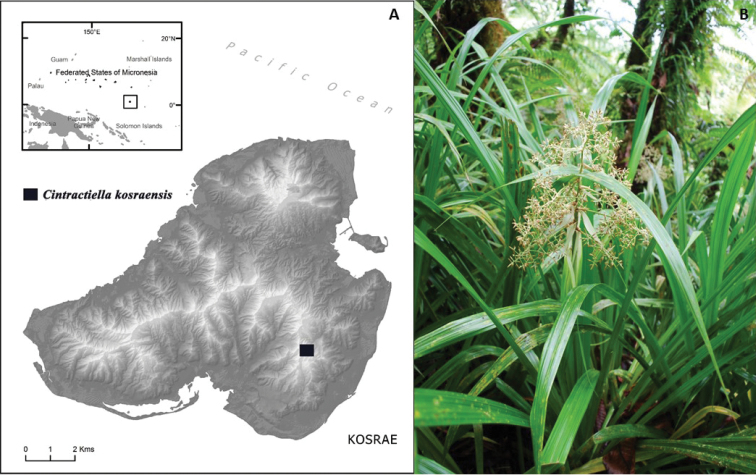
**A** Type location of *Cintractiellakosraensis* on the island of Kosrae, Federated States of Micronesia **B** The indigenous host, *Mapaniapacifica*, occurring along the summit ridge to Mt Oma.

Field surveys for botanical specimens were conducted on the island of Kosrae (5°20'N and 163°0'E) in September 2009. Due to the extreme steepness, inaccessibility and thickness of vegetation within this study region, survey transects were chosen intuitively and conformed to regional contours that were safely approachable. Herbarium voucher collections have been made in order to document common and rare plant taxa and for species identifications. Data for plant specimen vouchers are entered into the National Tropical Botanical Garden (PTBG) herbarium database. Specimens are being curated primarily at the Bishop Museum (BISH) and PTBG herbaria. Photographs of plants and habitats are curated by the NTBG and stored within a digital asset management system (i.e. ResourceSpace). The NTBG maintains a checklist of vascular plant taxa observed within the study region (Microsoft Excel database). Latitude and longitude coordinates were recorded by a Garmin GPSmap 60CSx (Garmin corp., Olathe, Kansas, U.S) unit in Lat/Long decimal for herbarium specimen data. The new smut species was found along the summit ridge of Mt. Oma in Malem Municipality (Fig. 1A) on the indigenous sedge *M.pacifica* (Fig. 1B). Materials studied here were deposited in the Kriebel Herbarium (PUL) and National Tropical Botanical Garden (PTBG).

Spores were mounted in lactic acid in glycerol. Light microscopic analyses were performed using a Nikon Eclipse 80i microscope (Nikon corp., Tokyo, Japan). Photomicrographs were obtained with a DS-Fi1 Nikon camera. Measurements are of a minimum of sixty randomly selected spores.

## Taxonomy

### Cintractiella kosraensis

Taxon classificationFungiUstilaginalesCintractiellaceae

Aime, M.Abbasi & K.R.Wood sp. nov.

MycoBank No: MB826716

Fig. 2

#### Diagnosis.

Differs from the similar *Cintractiellalamii* in having thin walled mostly depressed-globose spores with no visible germ pore and in lacking the hard, cylindrical curved mass of spores and hypertrophic parenchymatic tissue on the leaves, characteristic of *C.lamii*.

#### Type.

CAROLINE ISLANDS: The State of Kosrae: Malem Municipality, Mount Oma, 410 m alt., on *Mapaniapacifica* (Hosok.) T. Koyama, 4 Sep 2009, K.R.Wood 13895 (holotype: PTBG-070102; isotype: PUL F2910).

#### Description.

**Figure 2.**
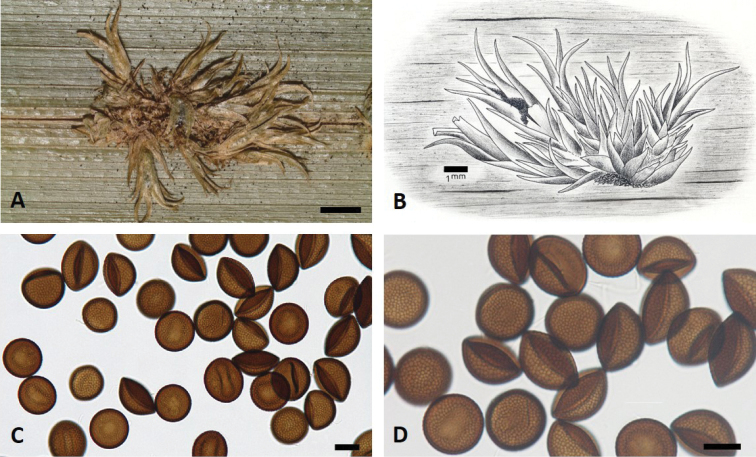
*Cintractiellakosraensis* (holotype, PTBG-070102) **A–B** sori on leaf as a photomicrograph (**A** Scale bar: 2 mm) and a line drawing (**B** Scale bar: 1 mm). **C–D** teliospores (Scale bars: 25 µm).

Sori amphigenous, mostly hypophyllous, clustered in groups of spikelets, each composed of olivaceous-brown, scale-like leaves, 1–1.5 mm wide, up to 6 mm long (Fig. 2A–B). Spore mass black, initially agglutinated and surrounded by a thin hyaline membrane, with no hard cylindrical body; at maturity, exposed at the opened tip of the spikelet. Spores single, mostly depressed-globose, globose or semi-globose, (28–) 35–44 µm in diameter, with no visible germ pore, wall dark reddish-brown, (1.2–) 1.5–2.5 (–3) µm thick, minutely reticulate (Fig. 2C–D). Spore germination not known.

#### Distribution and ecology.

*Cintractiellakosraensis* sp. nov. is only known from the type location along the summit ridge of Mt. Oma in Malem Municipality and type host–the indigenous sedge *M.pacifica*–on the volcanic island of Kosrae, located in the eastern Caroline Islands and within the Federated States of Micronesia in the general vicinity of 5°20'N, 163°0'E (Lorence and Wood 2012, Figure 1A).

#### Etymology.

*kosraensis* = for the island of Kosrae, where this species was discovered.

#### Specimens examined.

Caroline Islands. The State of Kosrae: Malem Municipality, Mount Oma, 410 m alt., on *M.pacifica*, 4 Sep 2009, K.R.Wood 13895 (holotype: PTBG-070102; isotype: PUL F2910).

## Discussion

*Cintractiella* is an unusual genus amongst smut fungi that produces sori in adventitious spikelets on vegetative or generative organs of members of tribe *Hypolytreae* (subfam. Mapanioideae, Cyperaceae). Only two other species have been described: *C.diplasiae* and *C.lamii*. *Cintractielladiplasiae* differs from *C.kosraensis* in producing sori in the host inflorescences and also producing teliospores with walls covered by blunt, rather densely situated, rarely confluent warts of variable sizes (Vánky 2003). *Cintractiellalamii* produces masses of teliospores in “peculiar galls”, i.e. adventitious branches with scale-like leaves, growing out of hypertrophic parenchymatic tissue on the abaxial side of the lamina of leaves, similar to *C.kosraensis*. However, in *C.lamii*, these are agglutinated and protrude as a column from the tips of the branches, whereas no column is formed in *C.kosraensis*. Teliospores are also diagnostic: in *C.lamii* these are globose, more or less flattened at one side, 29–36 μm and dark brown with a germpore and spore walls that are 3–4 μm thick and finely reticulate (Piepenbring 2001).

All three known members of *Cintractiella* parasitise members of Mapanioideae in Cyperaceae. The only report of *C.lamii* is from *Hypolytrum* sp. from Indonesia; *C.diplasiae* is found on *Diplasiakarataefolia* in Brazil, Trinidad and Venezuela (Vánky 2003). To our knowledge, *C.kosraensis* is the first smut fungus known to infect a species of *Mapania*.

Ideally, the description of new taxa is supported by abundant material from multiple collections. However, especially when considering microfungi from remote locales, these optima often cannot be met. Nonetheless, description of new species, even from limited material, adds to our understanding of fungal diversity (Kurtzman 2010) and highlights regions and lineages for which in-depth studies are needed. Most of the South Pacific islands remain under-explored for fungi, although these also appear rich in rare and endemic taxa (e.g. Kijpornyongpan and Aime 2016). Importantly, newly discovered taxa from rare lineages were shown to harbour the majority of novel genes in comparative genomic studies in smut fungi (Kijpornyongpan et al. 2018), highlighting the urgency in documenting this diversity before it disappears.

## Supplementary Material

XML Treatment for Cintractiella kosraensis

## References

[B1] Boedijn KB (1937) A smut causing galls on the leaves of *Hypolytrum*. Bulletin du Jardin Botanique de Buitenzorg, Serie 3(14): 368–372.

[B2] Costion CM Lorence DH (2012) The Endemic Plants of Micronesia: A Geographical Checklist and Commentary. Micronesica 43: 51–100.

[B3] Duncan RA Clague DA (1985) Pacific plate motion recorded by linear volcanic chains. In: Nairn AEM Stehli FG Uyeda S (Eds) The Ocean Basins and Margins, Vol.7A, The Pacific Ocean. Plenum Press, New York, 89–121. 10.1007/978-1-4613-2351-8_3

[B4] Kijpornyongpan T Aime MC (2016) Rare or rarely detected? *Ceraceosorusguamensis* sp. nov.: A second described species of Ceraceosorales and the potential for underdetection of rare lineages with common sampling techniques. Antonie van Leeuwenhoek 109: 1127–1139. 10.1007/s10482-016-0715-4 27236321

[B5] Kijpornyongpan T Mondo SJ Barry K Sandor L Lee J Lipzen A Pangilinan J LaButti K Hainaut M Henrissat B Grigoriev IV Spatafora JW Aime MC (2018) Broad genomic sampling reveals a smut pathogenic ancestry of the fungal clade Ustilaginomycotina. Molecular Biology and Evolution 35(8): 1840–1854. 10.1093/molbev/msy072 29771364

[B6] Kurtzman C (2010) Description of new yeast species–is one strain enough? The Bulletin of BISMiS 2010: 17–24.

[B7] Law W, Chellew M, Wood KR, Lorence DH, Cianchini C, Sanney J (in prep) Endangered Endemic Plants of Kosrae, Federated States of Micronesia.

[B8] Lorence DH Flynn T (2009) Checklist of the Plants of Kosrae, Federated States of Micronesia. Technical Report, National Tropical Botanical Garden.

[B9] Lorence DH Wood KR (2012) *Psychotriakosraensis* (Rubiaceae), A New Species from Kosrae, Caroline Islands, Micronesia. Novon 22: 51–55. 10.3417/2011048

[B10] Piepenbring M (2001) *Cintractielladiplasiae* – a second species of *Cintractiella* (Ustilaginales) with sori in adventitious spikelets on Hypolytreae (Cyperaceae). Perspectives in Plant Ecology, Evolution and Systematics 4: 116–120. 10.1078/1433-8319-00018

[B11] Vánky K (2003) Cintractiellaceae fam. nov. (Ustilaginomycetes). Fungal Diversity 13: 167–173.

[B12] Vánky K (2013) Illustrated Genera of Smut Fungi (3^rd^ edn). APS Press, St. Paul, MN, 288 pp.

